# Tetrameric glycoprotein complex gH/gL/gQ1/gQ2 is a promising vaccine candidate for human herpesvirus 6B

**DOI:** 10.1371/journal.ppat.1008609

**Published:** 2020-07-23

**Authors:** Bochao Wang, Kouichi Hara, Akiko Kawabata, Mitsuhiro Nishimura, Aika Wakata, Lidya Handayani Tjan, Anna Lystia Poetranto, Chisato Yamamoto, Yasunari Haseda, Taiki Aoshi, Lisa Munakata, Ryo Suzuki, Masato Komatsu, Ryuko Tsukamoto, Tomoo Itoh, Chikako Nishigori, Yasuyuki Saito, Takashi Matozaki, Yasuko Mori

**Affiliations:** 1 Division of Clinical Virology, Center for Infectious Diseases, Kobe University Graduate School of Medicine, Kobe, Hyogo, Japan; 2 Vaccine Dynamics Project, BIKEN Innovative Vaccine Research Alliance Laboratories, Research Institute for Microbial Diseases, Osaka University, Suita, Osaka, Japan; 3 BIKEN Center for Innovative Vaccine Research and Development, The Research Foundation for Microbial Diseases of Osaka University, Suita, Osaka, Japan; 4 Laboratory of Drug and Gene Delivery Research, Faculty of Pharma-Science, Teikyo University, Itabashi-ku, Tokyo, Japan; 5 Department of Diagnostic Pathology, Kobe University Hospital, Kobe, Hyogo, Japan; 6 Division of Dermatology, Department of Internal Related, Kobe University Graduate School of Medicine, Kobe, Hyogo, Japan; 7 Division of Molecular and Cellular Signaling, Department of Biochemistry and Molecular Biology, Kobe University Graduate School of Medicine, Kobe, Hyogo, Japan; Emory Vaccine Center, UNITED STATES

## Abstract

Primary infection of human herpesvirus 6B (HHV-6B) occurs in infants after the decline of maternal immunity and causes exanthema subitum accompanied by a high fever, and it occasionally develops into encephalitis resulting in neurological sequelae. There is no effective prophylaxis for HHV-6B, and its development is urgently needed. The glycoprotein complex gH/gL/gQ1/gQ2 (called 'tetramer of HHV-6B') on the virion surface is a viral ligand for its cellular receptor human CD134, and their interaction is thus essential for virus entry into the cells. Herein we examined the potency of the tetramer as a vaccine candidate against HHV-6B. We designed a soluble form of the tetramer by replacing the transmembrane domain of gH with a cleavable tag, and the tetramer was expressed by a mammalian cell expression system. The expressed recombinant tetramer is capable of binding to hCD134. The tetramer was purified to homogeneity and then administered to mice with aluminum hydrogel adjuvant and/or CpG oligodeoxynucleotide adjuvant. After several immunizations, humoral and cellular immunity for HHV-6B was induced in the mice. These results suggest that the tetramer together with an adjuvant could be a promising candidate HHV-6B vaccine.

## Author summary

Human herpesvirus 6B (HHV-6B) is known as the cause of the common childhood febrile illness exanthem subitum in its primary infection, and it develops into a lifelong latent infection in almost all individuals. Severe complications such as meningitis and encephalitis can occur in both the primary infection and reactivation. There is no established treatment or vaccine. The tetrameric glycoprotein complex gH/gL/gQ1/gQ2 (tetramer) on the viral envelope is the ligand for the entry of HHV-6B, which is the critical part for its infection. Here, we established a soluble form of the tetramer and purified it to homogeneity. After several immunizations of tetramer along with different combinations of adjuvants in mice, we observed that it greatly induced defensive immunity against HHV-6B, indicating that the tetramer has the potential to become a vaccine candidate. Moreover, our results also revealed that combinations of distinct adjuvants with the tetramer would be useful as an HHV-6B vaccine strategy for different purposes.

## Introduction

Human herpesvirus 6B (HHV-6B) infects infants during the window of susceptibility after a decline of maternal immunity, usually at the ages 6–18 months. This primary infection causes exanthema subitum with a symptom of fever followed by skin rash (*roseola*), and in the worst cases, it develops into encephalitis leaving neurological sequelae[1,2,3,4]. HHV-6B establishes life-long latency after the primary infection, as do other members of the human herpesvirus family. The latent virus is able to reactivate, and due to host immunity, it usually does not manifest as any symptoms. However, HHV-6B infection is clinically problematic in immunocompromised adults undergoing stem cell or an organ transplantation, particularly umbilical cord blood transplantation as a treatment for leukemia. HHV-6B reactivation occurs after transplantation, and it leads to viremia and life-threating encephalitis at a significantly high rate, 10%–20% [5]. The prevalence of HHV-6B infection is extremely high; >90% adults are seropositive (>99% in Japan) [6]. Thus, almost all humans are infected with and carry HHV-6B. There is no effective prophylaxis for HHV-6B, and the establishment and maintenance of immunity against HHV-6B is therefore necessary for human health.

Envelope glycoproteins of herpesviruses play crucial roles in the initiation of infection from the extracellular environment. Glycoprotein B (gB) and a hetero-dimeric complex of glycoprotein H and L (gH/gL) are common viral proteins for the herpesvirus family. gB is a class III fusion protein that directly catalyzes the fusion event between the viral membrane and the host cell membrane [7]. gB is not constantly active, and its function is regulated by the gH/gL complex [8]. Effective access to a cognate target cell requires additional glycoproteins to achieve the receptor-mediated infection. Members of the herpesvirus family possess respective viral ligands to host receptors. For HHV-6A and HHV-6B, they are gO and gQ1/gQ2 [9]. gH/gL of HHV-6B is combined with gQ1/gQ2 or gO in the trafficking pathway through the trans-Golgi network, resulting in a hetero-tetramer (gH/gL/gQ1/gQ2) and a trimer (gH/gL/gO), respectively [10,11,12,13]. The gH/gL/gQ1/gQ2 complex, referred to hereafter as 'tetramer,' is especially important because gQ1 associated with gQ2 is the determinant of cell tropism [14]. Although the tetramer of HHV-6A recognizes the ubiquitously expressed host receptor CD46 [15,16], the HHV-6B tetramer interacts with a different receptor, i.e., hCD134, expressed specifically in activated T cells [17,18,19].

Envelope glycoproteins are major targets of host cell immunity because of their outermost location on the virion and their critical functions for infection. The immunization of mice with a UV-irradiated virion elicited monoclonal antibodies with neutralizing activity against HHV-6A and/or HHV-6B [20,21,22,23,24]. The target proteins of these antibodies have been identified as glycoproteins including gB [22], gH [20,23,25], and gQ1 [21,24,26]. We observed high antigenicity of gQ1 in our previous research, showing that most (five of the six) obtained anti-gQ1 antibodies have neutralizing activity against HHV-6B [24]. An anti-gQ1 antibody named KH-1 specifically recognizes and potently neutralizes HHV-6B, and the epitope depends on the conformation of gQ1 associated with gQ2, suggesting the importance of the conformation of gQ1 in the context of the tetramer.

In our recent research, we also identified the CD4^+^ and CD8^+^ T-cell epitopes of gQ1 in BALB/c mice by using *in vivo* electroporation with a plasmid DNA encoding this protein [27]. These findings motivated us to develop a subunit vaccine based on the tetramer. Since gQ1 is responsible for the receptor-mediated infection via hCD134 on T cells, antibodies elicited against gQ1 are expected to interrupt the engagement between the viral ligand and the host receptor.

In contrast to several reports of the development of vaccines against other herpesviruses, there are no published studies describing the development of a vaccine against HHV-6B despite its high clinical burden. We conducted the present study to analyze the potency of HHV-6B tetramer to induce immunity. An expression and purification system of the soluble tetramer was established. Purified protein was administered to mice with adjuvants including the widely used aluminum hydroxide gel adjuvant (Alum) and D35, which belongs to the group of CpG oligodeoxynucleotide adjuvants, which showed advantages in inducing cellular immunity by stimulating the innate immune receptor, toll-like receptor 9 (TLR9) [28,29]. Our analyses of both humoral and cellular immunity corroborated the effectiveness of the use of the tetramer as a prophylactic subunit vaccine.

## Results

### Expression and purification of soluble HHV-6B tetramer

**Fig 1:**
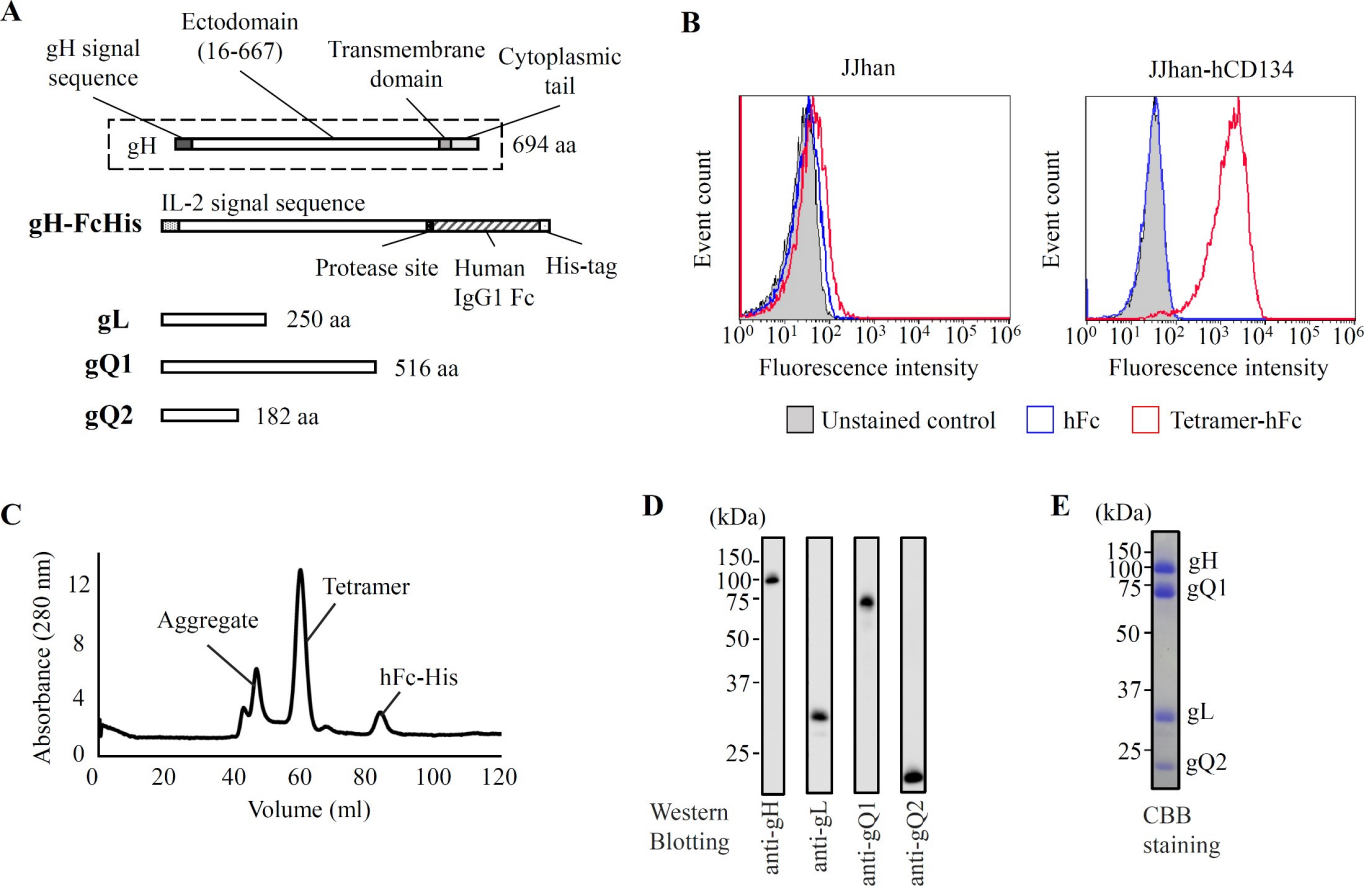
Expression and purification of the recombinant gH/gL/gQ1/gQ2 complex. **(A)** The constructions of gH, gL, gQ1, and gQ2 are shown. gH is modified to have an N-terminal IL-2 signal sequence and a C-terminal human IgG1 Fc (hFc) and His_6_ sequence replacing the intrinsic gH signal sequence and transmembrane-cytoplasmic tail domains, respectively. (**B)** Cell binding assay was performed using hCD134 expressing JJhan cells or JJhan cells (negative control). After incubation with the tetramer-hFc containing medium, the interaction between cell expressing hCD134 and tetramer-hFc was detected using Alexa 488 conjugated anti-human IgG targeting the hFc by flow cytometry. The hFc protein (without tetramer) was used as the non-binding negative control. (**C)** Size exclusion column chromatography of the purified tetramer. (**D)** Western blotting analysis of the tetramer purified by the size exclusion column chromatography. gH, gQ1, gL, and gQ2 were detected by the respective antibodies. (**E)** Purified gH/gL/gQ1/gQ2 complex detected by Coomassie brilliant blue staining.

To exploit the HHV-6B tetramer as a subunit vaccine, we constructed an expression system for the recombinant tetramer. Because the tetramer is tethered on the membrane via a single transmembrane domain within gH, we designed its soluble form by deleting the transmembrane domain of gH (Fig 1A). For the facilitation of the expression and purification, an interleukin (IL)-2 signal sequence (IL-2ss) and a human IgG1 Fc (hFc; 227 amino acids) tag with His_6_ sequence were attached as replacements of the N-terminal intrinsic signal sequence and the C-terminal transmembrane-cytoplasmic domain of gH, respectively (Fig 1A).

Two plasmids, one containing gH and gL genes and the other containing gQ1 and gQ2 were co-transfected into HEK293S GnTI^−^ cells, and a stable expression cell line was established under the selection of antibiotics. The receptor binding activity of the tetramer secreted into the cell culture medium was certified by flow cytometry, and the tetramer binding to the cells which express the receptor (hCD134) were demonstrated (Fig 1B) [17,30]. To purify the tetramer to homogeneity, we first collected the secreted protein by using the Ni-chelating affinity via the His_6_-tag at the C-terminus of recombinant gH. After elution, hFc and His_6_ tags were removed by protease using the designed protease site (Fig 1A). Further purification was performed by size exclusion chromatography. The tetramer was eluted as a single peak separated from the aggregate peak and the hFc peak (Fig 1C). The purified tetramer in the peak contained all four subunits (Fig 1D), indicating the complex formation. No obvious contamination was observed (Fig 1E).

### Humoral immunity induced by tetramer vaccination

**Fig 2:**
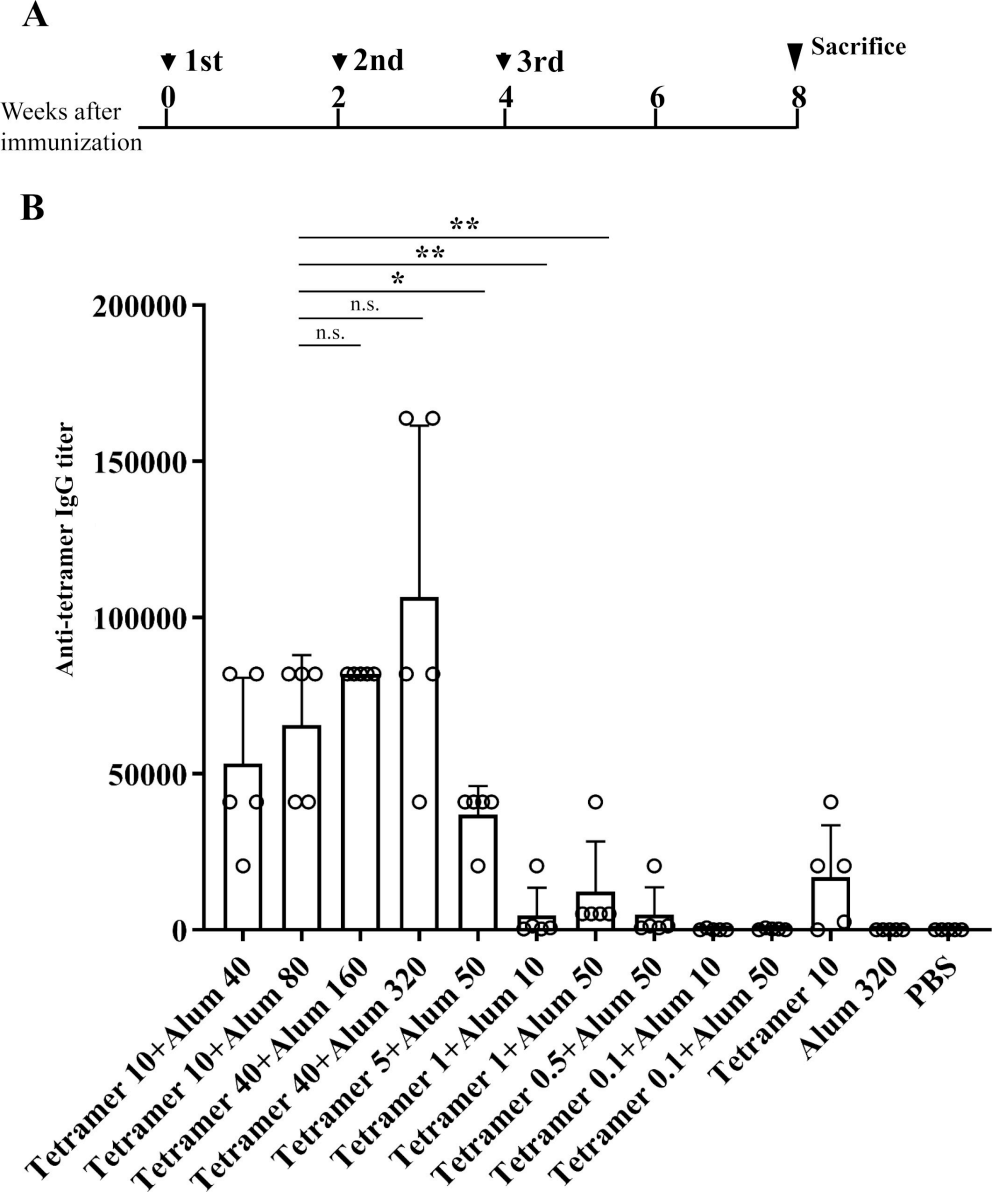
Humoral immunity induced by immunization with different amounts of the tetramer and Alum. **(A)** Immunization schedule indicated as the weeks from the fist immunization of the four-week-old female BALB/c mice (n = 5). (**B)** The amounts of the anti-tetramer antibodies in the sera collected from the immunized mice at the eight weeks after the third immunization as indicated in (A) were evaluated by the ELISA assay using purified tetramer as the antigen. The titers were calculated as the maximum dilution at which the value of OD405 was higher than that of the mean+2SD of the group immunized with PBS. *p<0.05, **p<0.01 by Student's t-test. n.s.: not significant.

First, to determine the immunogenicity of the tetramer, we mixed different amounts of purified protein with Alum and subcutaneously injected the mixture three times into 4-, 6-, and 8-week-old BALB/c mice (Fig 2A). The sera of the mice were collected at four weeks after third immunization, and the titer of anti-tetramer antibodies was analyzed by an ELISA assay (Fig 2B). Generally, a reduction in the amount of either the tetramer or the Alum dose-dependently reduced the antibody titers. The administration of 40 μg of the tetramer with 320 μg of Alum induced a high titer of antibodies. A reduced amount of tetramer, 10 μg, resulted in a slightly decreased antibody titer in a manner that depended on the dose of Alum (40 μg and 80 μg), although this trend was not significant when the amount of tetramer was over 10 μg. A significant decrease in the antibody titer was detected when mice were immunized with <5 μg of the tetramer. Immunization with 10 μg of the tetramer without Alum induced low antibody titers.

Alum is known as an adjuvant that induces mainly humoral immunity as its response to T helper 2 (Th2) cells [31]. To induce the cellular immunity which might be important for the defense against herpesvirus reactivation, we also mixed the tetramer with D35 (one of the A-class CpG oligodeoxynucleotide adjuvants) which showed great activity in inducing cellular immunity with several viral proteins in recent research [32]. Cationic liposomes have also been used as efficient antigen delivery systems. Combinations of N-[1-(2,3-Dioleoyloxy) propyl]-N,N,N-trimethylammonium methylsulfate (DOTAP) and D35 have been shown to induce an enhanced production of IFN-α in several cases [28,32].

**Fig 3:**
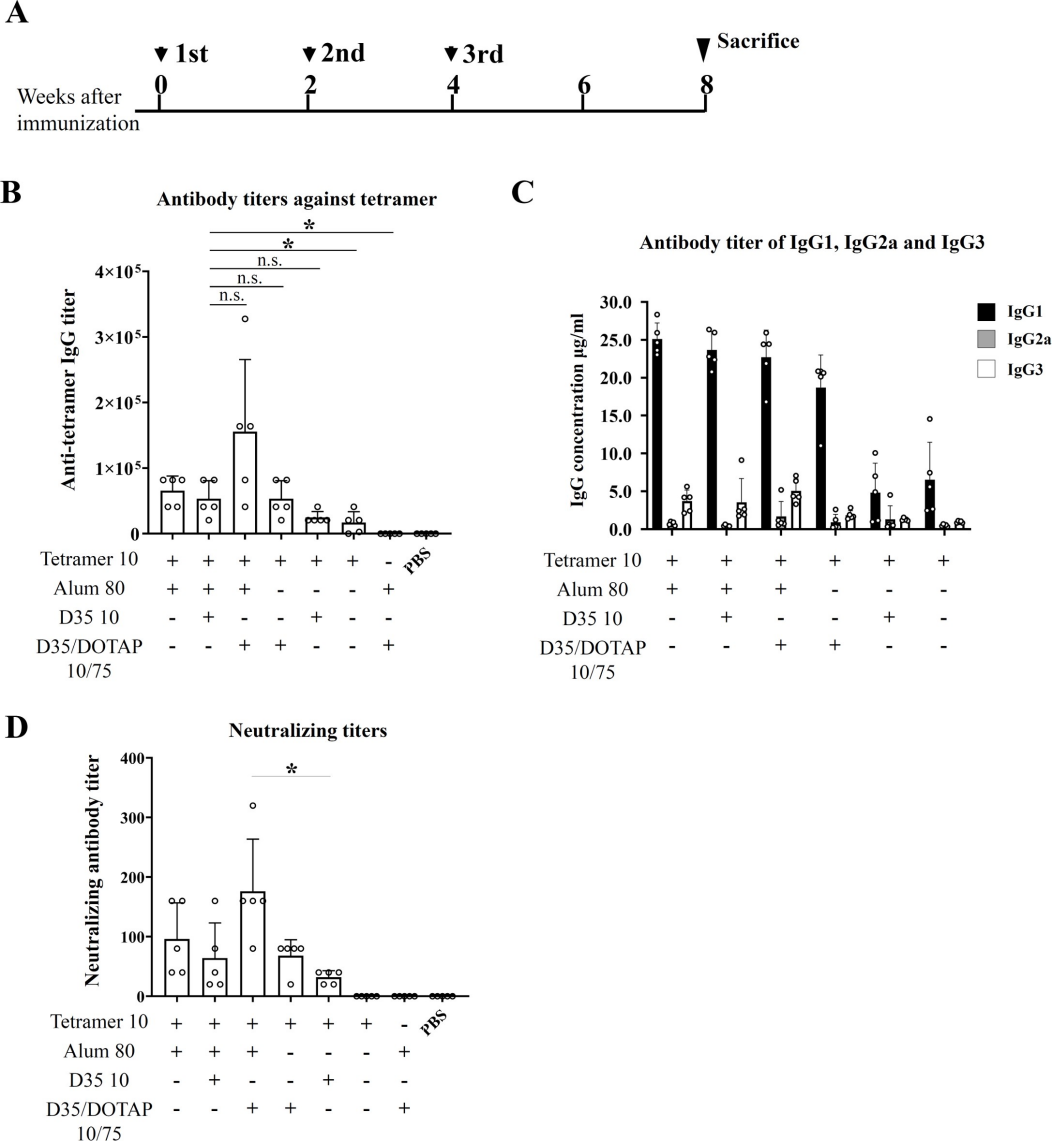
Humoral immunity induced by immunization with the tetramer and the combination of Alum and CpG adjuvant. **(A)** Immunization schedule indicated as the weeks of immunization of four-week-old female BALB/c mice (n = 5). (**B)** The amounts of the anti-tetramer antibodies in the sera collected from mice immunized with the indicated combination of adjuvants at the eight weeks after the third immunization were evaluated for the further ELISA assay using purified tetramer. The titers were calculated as the maximum dilution at which the value of OD405 was higher than that of the mean+2SD of the group immunized with PBS. (**C)** The IgG isotypes of the anti-tetramer antibodies were analyzed by the ELISA assay at the dilution of 2560. IgG1, 1gG2a and IgG3 were detected separately from the same sera. The values of serum estimated IgG concentration were obtained from standard curves for the isotype control antibodies of mouse IgG1, IgG2a and IgG3, and presented as the mean±SD. (D) The neutralizing activities of the sera from the immunized mice indicated were measured in MT4 cells. Titers are presented as the maximum dilution at which the HHV-6B IE1 protein could not be detected. Data are the mean±SD of each group. *p<0.05 by Student's t-test. n.s.: not significant.

Based on our present findings using Alum, we determined the appropriate amount of the tetramer as 10 μg per mouse, along with 80 μg alum as the standard. We also used 10 μg D35 complexed with or without 75 μg DOTAP for immunization. BALB/c mice (n = 5/group) were immunized subcutaneously at intervals and sacrificed at the same schedule (Fig 3A). The ELISA assay using the mouse sera revealed that the highest titer of anti-tetramer antibodies was achieved from the group immunized with a combination of Alum, D35, and DOTAP (Fig 3B). No significant difference was detected between the groups immunized with tetramer + Alum or with tetramer + D35/DOTAP, but both of these groups showed significantly induced antibody titers compared to the immunization with tetramer without any adjuvants or with tetramer + D35 alone (Fig 3B).

To further characterize the antibodies elicited against the tetramer, we analyzed the anti-tetramer titers of IgG1, IgG2a and IgG3 from the same sera in the Fig 3B. As shown in Fig 3C, immunization of the tetramer with all of the tested combinations of adjuvants and tetramer alone dominantly induced IgG1 antibodies.

### Antibody titer of human sera against HHV-6B tetramer

We wondered whether HHV-6B infection in human would naturally produce the antibodies against tetramer which would be important for our vaccination strategy. We examined the antibody titers against the HHV-6B infected cells or the tetramer for the sera from 14 patients of drug-induced hypersensitivity syndrome (which is highly related with HHV-6 reactivation) and 5 healthy adults (Table 1). With one exception, all the sera reacted to the tetramer as well as the HHV-6B infected cells (S1 Fig). The antibody titer against tetramer showed similar tendency to those against the HHV-6B infected cells.

**Table 1:** Antibodies titers of human sera against HHV-6B infected cells and tetramer.

No.*	Titer (infected cells)^†^	Titer (tetramer)^‡^
1	640	160
2	640	160
3	1280	80
4	40	<10
5	320	20
6	160	40
7	320	160
8	40	40
9^§^	640	80
10	160	40
11	160	20
12	80	80
13	80	20
14^§^	16000	2560
A	80	40
B	320	40
C	80	80
D	80	80
E	320	80

*No. 1–14 indicated the sera from 14 patients of drug-induced hypersensitivity syndrome (DIHS) and No. A-E indicated the sera from five health volunteers.

† Reciprocal of the endpoint dilution in the IFA assay.

‡ Reciprocal of the endpoint dilution in the ELISA assay.

§ HHV-6 DNA was detected in the serum by nested-PCR.

### Neutralizing antibodies induced by the tetramer vaccination

The neutralization potency against *in vitro* HHV-6B infection of the collected sera checked in Fig 3 was analyzed. The neutralizing titer was evaluated as the dilution rate of the serum at which the HHV-6B immediate early protein 1 (IE1) was not detected in MT4 cells subjected to HHV-6B infection. The mean titers of each group are illustrated in Fig 3D. Though the anti-tetramer antibody titer of the group immunized with 10 μg of the tetramer without adjuvants was the same as that of the group immunized with D35 alone, its neutralizing titer was the lowest among all of the groups immunized with the tetramer, as it was under the minimum dilution rate in this experiment.

However, the highest titer of neutralizing antibodies was detected from the group immunized with tetramer + Alum and D35/DOTAP, which is related to its highest titer against the tetramer (Fig 3B). The groups immunized with tetramer + Alum with or without D35 showed no difference in the neutralizing titers. The mice immunized with tetramer + D35/DOTAP showed a significant increase in neutralizing titer compared to the group immunized with tetramer + D35 alone.

### Cytokine secretion induced by the stimulation of the tetramer or a gQ1-specific peptide

**Fig 4:**
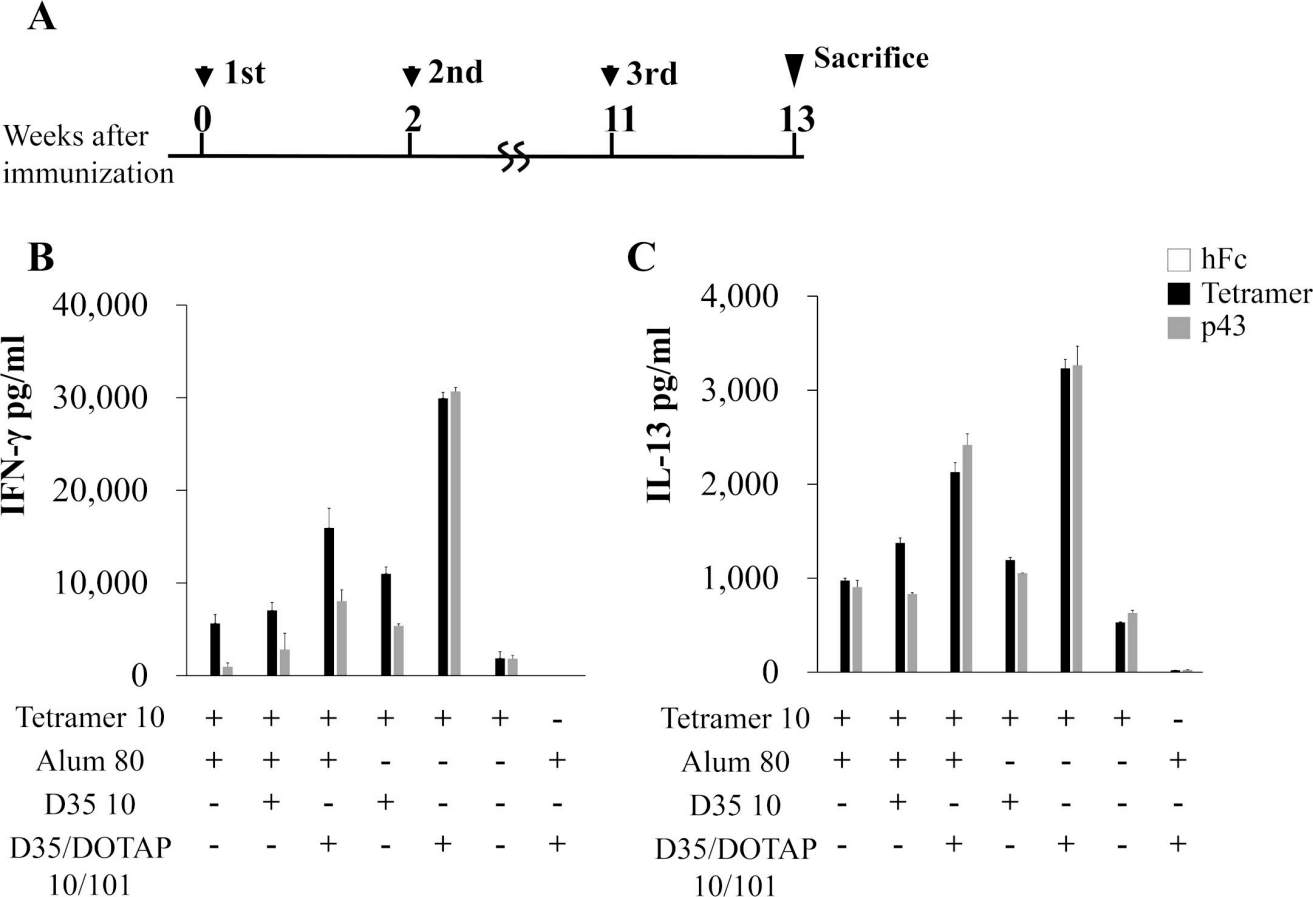
Cellular immunity induced by immunization with the tetramer. **(A)** Immunization schedule indicated as the weeks of immunization of four-week-old female BALB/c mice (n = 5). At the 13 weeks after the third immunization, single cell suspensions were prepared from the mouse spleens after the hemolysis of red blood cells. The productions of IFN-γ (**B**) and IL-13 (**C**) in the supernatant in response to the stimulation of indicated proteins were measured by ELISA assays.

As the cellular immunity plays an important role for the protection as well as the humoral immunity, we also assessed the immune cell response against the tetramer by evaluating the secretions of interferon-gamma (IFN-γ) and IL-13 after stimulation with the tetramer and the peptide p43 which showed the potential to induce CD4^+^ T cell-mediated immunity [27]. BALB/c mice (n = 5/group) were immunized at the schedule shown in Fig 4A. Lymphocytes from the spleen were stimulated with the hFc, the tetramer, or p43. The IFN-γ secretion after the tetramer stimulation showed a pattern that was similar to that of the antibody titers described above (Fig 4B).

A notable difference is that the group immunized with D35/DOTAP without Alum showed the highest secretion of IFN-γ. The group immunized with all of the adjuvants also showed high levels of IFN-γ. The results of the p43 stimulation indicated a pattern that was similar to that achieved with the tetramer. For IL-13, which is secreted mainly by Th2 cells, the results of both the tetramer stimulation and p43 stimulation showed patterns that were similar to that of IFN-γ secretion (Fig 4C).

**Fig 5:**
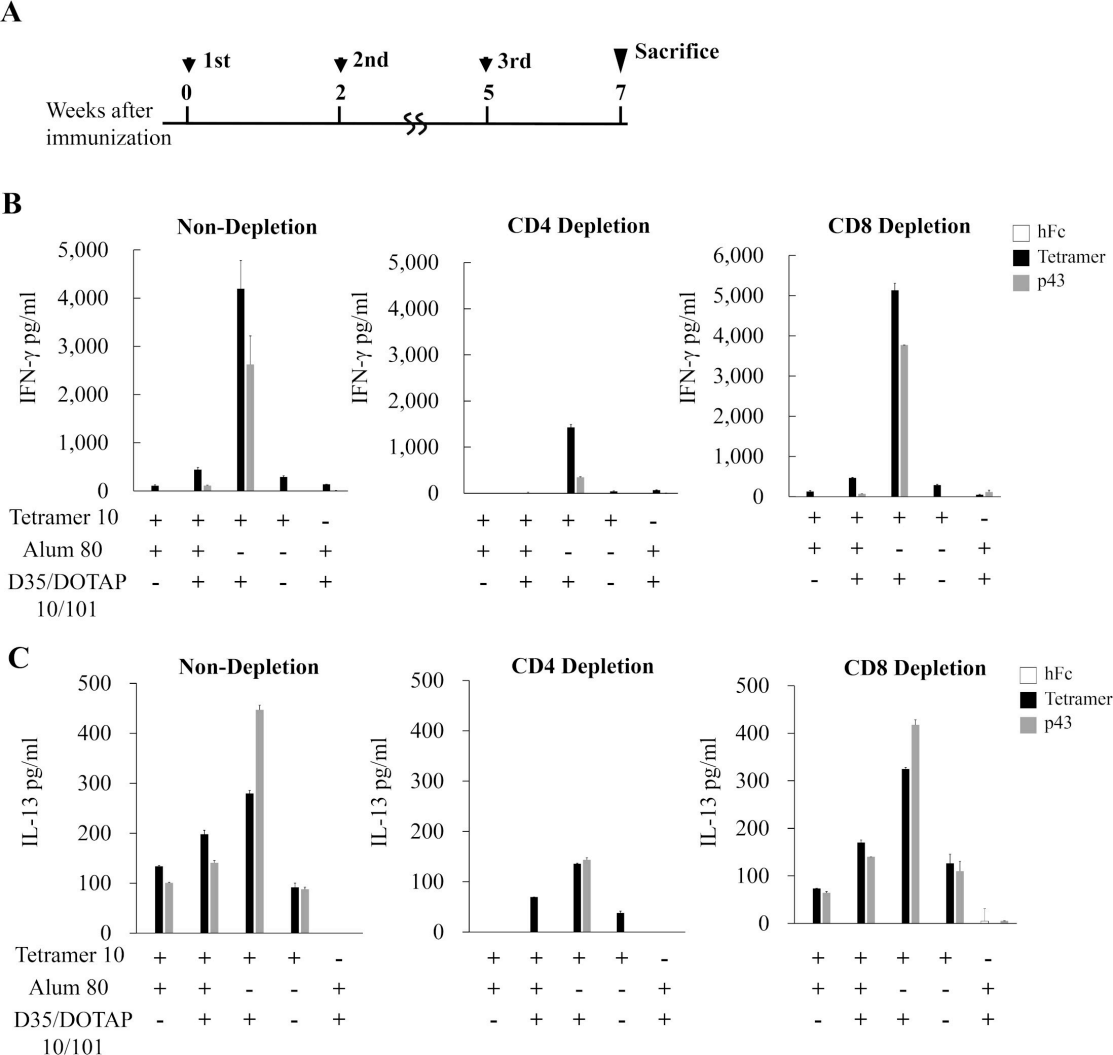
Cell type responsible for the cytokine induction. **(A)** Immunization schedule indicated as the weeks of immunization of four-week-old female BALB/c mice (n = 3). At the seven weeks after the third immunization, single cell suspensions were prepared from the mouse spleens after the hemolysis of red blood cells was conducted and continued for the depletion of the indicated cell type. The productions of IFN-γ (**B**) and IL-13 (**C**) in supernatant were detected by ELISA assays.

To further determine the cell type which was responsible for the secretion of cytokines after stimulation, we performed an optional depletion of CD4^+^ or CD8^+^ T cells from the lymphocytes. BALB/c mice (n = 3/group) were immunized at the schedule shown in Fig 5A. The tetramer- or p43-induced IFN-γ was reduced in the CD4 depletion condition, but was little affected by the CD8 depletion (Fig 5B). A similar pattern was observed for IL-13, indicating that the major secretion of the cytokine was from the CD4^+^ T cells (Fig 5C).

### Protective effect of the tetramer-induced immunity in HHV-6B in vivo infection

HHV-6B exclusively infects human, and thus direct challenge experiment by using animals was practically impossible. As we recently established the first *in vivo* mouse model for HHV-6B infection [33], we employed the model to test the tetramer-induced immunity. Direct immunization to the humanized mice of the model was unfeasible as they based on immunocompromised mice, thus we examined the protecting effect of the tetramer-vaccinated sera.

**Fig 6:**
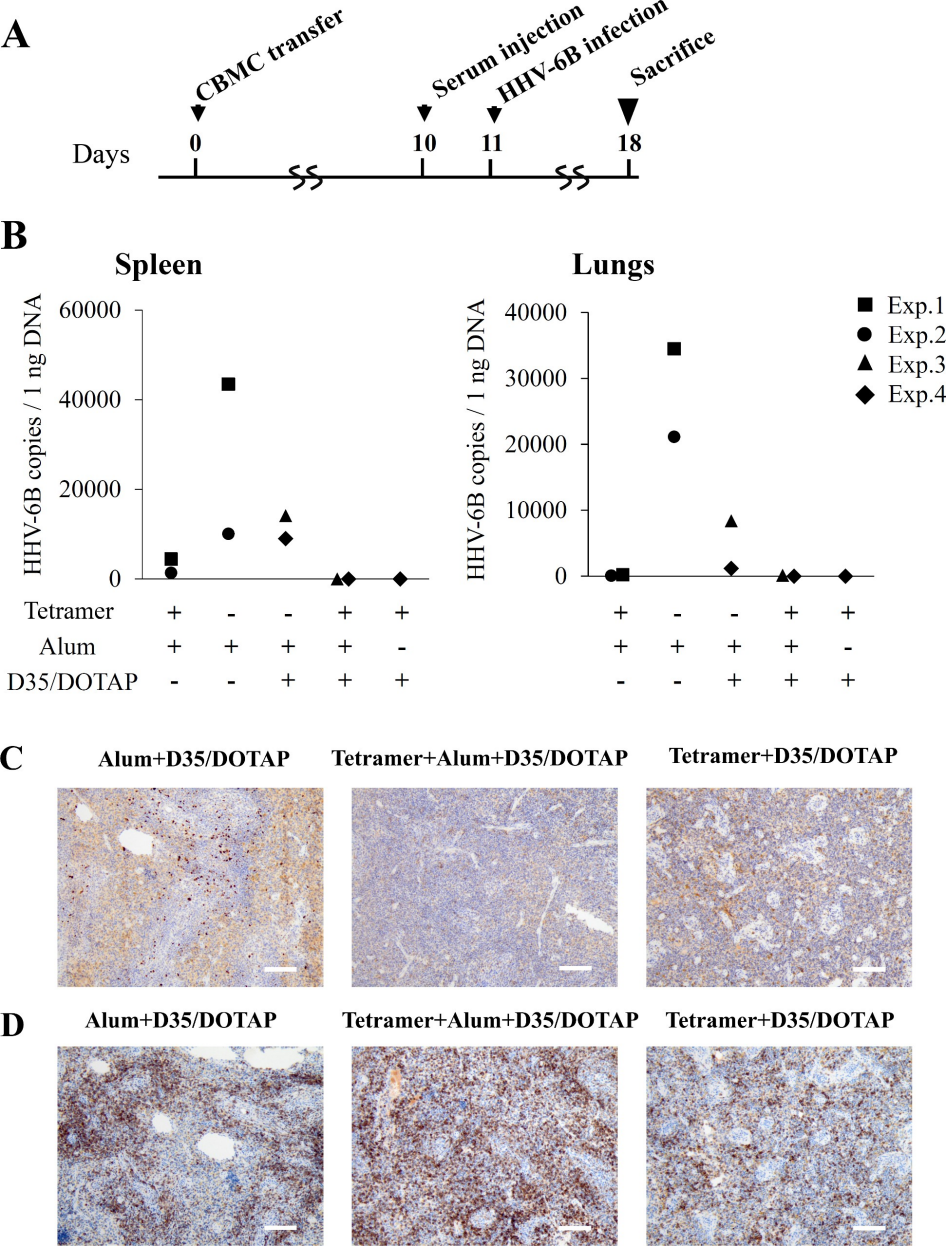
Humoral immunity induced by the vaccination prevented HHV-6B infection *in vivo*. **(A)** HHV-6B infection model was prepared by transferring human cord blood mononuclear cells (CBMCs) into humanized mice. After confirming the increase of human T cells, mice were pre-injected with the different kinds of sera. Sera from tetramer-immunized mice including tetramer+Alum, tetramer+Alum+D35/DOTAP or tetramer+D35/DOTAP, were used for the pre-injection. The sera from mice immunized with adjuvants only (Alum or Alum+D35/DOTAP) were also used as control. One humanized mouse was used for each group. Cell-free HHV-6B was infected intravenously into the mice, and the 50% tissue culture infective doses [TCID50]/ml of the HHV-6B in each experiment was as follows: Exp. 1: 1.4 × 10^5^; Exp. 2: 6.7 × 10^4^; Exp. 3: 9.6 × 10^4^; Exp. 4: 9.6 × 10^4^. HHV-6B-infected animals were euthanized at 7 days post-infection and the organs were collected for further analysis. (**B**) HHV-6B genome copies in the spleens and lungs were detected by quantitative PCR. Each of experiment 1–4 (Exp.1-4) indicated the experiment performed at the same infection timing. The expression of HHV-6B IE1 (**C**) and the infiltration of human CD3^+^ T cells (**D**) were detected by immunostaining of the spleen sections and shown as the representative photographs. Bar: 100 μm.

The pooled sera from two mice that were immunized three times with 40 μg tetramer together with 80 μg Alum (the neutralizing titers detected *in vitro* were both 320) or the pooled sera from two mice immunized three times with 320 μg Alum alone (the neutralizing titers were both <20) were used in the experiments one and two (Exp. 1 and Exp. 2). In the experiments three and four (Exp. 3 and Exp. 4), the following sera were used: the pooled sera from two mice immunized three times with 10 μg tetramer together with 80 μg Alum and 10 μg D35 complexed with 75 μg DOTAP (the neutralizing titers were 320); the serum from one mouse immunized three times with 10 μg tetramer together with 10 μg D35 complexed with 75 μg DOTAP (the neutralizing titer was 320); or the pooled sera from two mice immunized three times with all the adjuvants, 80 μg Alum and 10 μg D35 complexed with 75 μg DOTAP without the tetramer (the neutralizing titers were both <20). Sera were intravenously injected to the mice 1 day before HHV-6B infection (Fig 6A). Seven days later, all of the mice were sacrificed for viral detection. Lower virus loads in the spleens and lungs were detected from the tetramer immunized sera-injected groups compared to groups of Alum alone or Alum + D35/DOTAP by quantitative PCR (Fig 6B). Although Alum + D35/DOTAP group without tetramer showed a tendency of relatively lower viral load compared to Alum alone group, we did not focus on this difference in this study because of the limitation of available humanized mice. The immunostaining of the spleen sections also revealed lower expression levels of viral proteins in the mice injected with the tetramer immunized sera (Fig 6C) despite a similar or more infiltration of human T cells, which are the target of HHV-6B infection, compared to those of mice infused with the sera from the mice immunized with adjuvants only (Fig 6D).

### The tetramer induced cytokines/chemokines of monocyte-derived dendritic cells

**Fig 7:**
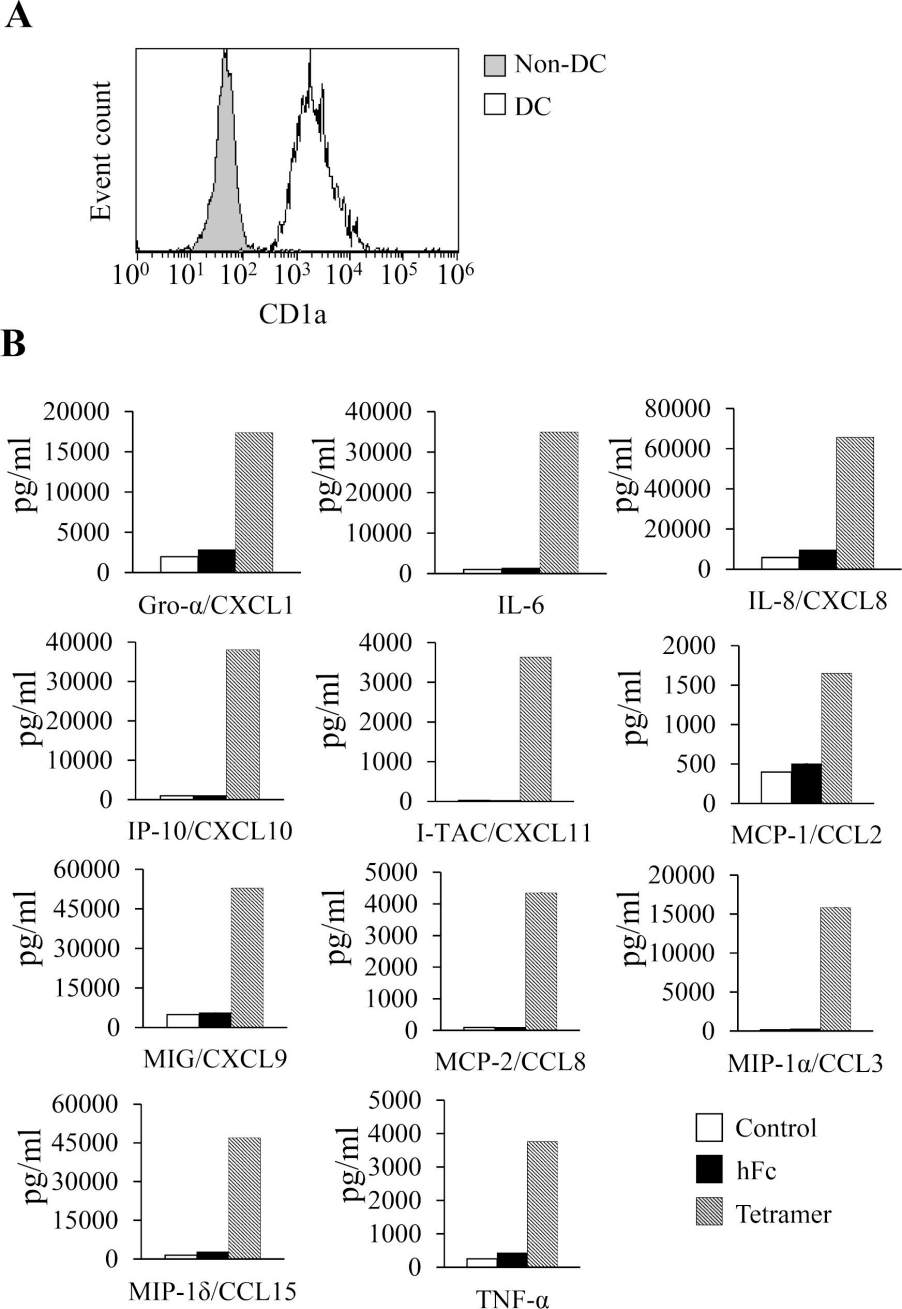
Human cytokine/chemokine levels in the culture supernatant of DCs treated with HHV-6B tetramer. (**A)** Detection of the marker of immature DC, CD1a, by flow cytometry to attest the prepared human monocyte derived DCs. (**B**) The secretions of cytokines/chemokines by human monocyte-derived DCs 24 hr after they were stimulated with culture medium (control), hFc, or tetramer were measured by a Bio-Plex system. The cytokines/chemokines that showed higher responses to the stimulation by the tetramer compared to the control and hFc are shown. Consistent results using cells from two donors were obtained, and the representative data of one volunteer are shown as the mean of duplicate measurements.

The above results (Figs 2 and 3) demonstrated that the tetramer without any adjuvants showed the ability to induce antibodies, although this ability was weaker compared to the groups in which adjuvants were used. We hypothesized that the tetramer itself might have the potential to induce innate immunity which could lead to acquired immunity stronger. Dendritic cells (DCs), which play an important role in the antigen presentation, might be stimulated by the tetramer, thus resulting in the secretion of cytokines/chemokines. To investigate this hypothesis, we made human monocyte-derived DCs from peripheral blood mononuclear cells (PBMCs) (Fig 7A). After stimulation with the tetramer, the cytokines/chemokines in the supernatant were analyzed by a multiplex cytokine/chemokine assay. The cytokines/chemokines induced with the tetramer whose concentrations were elevated compared to the control group stimulated with medium or the same amount of hFc were Gro-α/CXCL1, IL-6, IL-8/CXCL8, IP-10/CXCL10, I-TAC/CXCL11, MCP-1/CCL2, MCP-2/CCL8, MIG/CXCL9, MIP-1α/CCL3, MIP-1δ/CCL15, and TNF-α (Fig 7B).

## Discussion

Our analyses demonstrated immunity induced in mice by immunization with HHV-6B tetramer. With the use of the soluble form of the tetramer with an adjuvant, substantial humoral immunity was induced in the sera of the immunized mice, depending on the administration dose of the antigen with adjuvant. The sera of the mice contain anti-tetramer antibodies (Figs 2B and 3B), and they effectively prevented the *in vitro* and *in vivo* HHV-6B infection (Figs 3D and 6). The induction of immunity by the tetramer was substantially enhanced when the tetramer was used together with the Alum adjuvant and was slightly enhanced with the D35/DOTAP, although the tetramer itself had also weak potency for the induction.

Although the tetramer protein seems to be purified well (Fig 1C and 1E), the possibility of contamination in the antigen solution purified is thought to be an important issue for the vaccine development. We cannot rule out the possibility completely that the purified tetramer solution may contain some contaminant proteins and they might respond in the assays for the anti-tetramer antibody titer and for the T-cell response. However, the neutralizing antibody titer (Fig 3D) showed similar pattern with the anti-tetramer IgG titer (Fig 3B), implying that the anti-tetramer antibodies contributed to the neutralization. In the T-cell response analysis, the synthesized gQ1 peptide p43 [27] could also stimulate the T-cells (Fig 4 and Fig 5), thus the response was unlikely attributed to such contaminant proteins. The lack of T-cell response to the hFc (Fig 4 and Fig 5) indicated that such immunogenic contaminants were absent in the hFc solution which was produced and purified in the same procedure as the tetramer. The purity of the antigen would be addressed in the next stage of vaccine development as preclinical studies.

Most of the primary infection of HHV-6B occurred in children at 6–18 months old [6]. Hall et al. showed that primary infection occurs when the maternal antibody against HHV-6B is decreased [4]. It indicates that the maternal antibody, that is, humoral immunity can prevent the HHV-6B primary infection. Therefore, in the case of HHV-6B, it is considered to be effective to give the vaccination to infants in early stage of life from three months old as the DPT-IPV vaccine (D: diphtheria, P: pertussis, T: tetanus and IPV: inactivated poliovirus) which is regularly vaccinated in Japan [34].

Antibody titers against HHV-6B increase after the primary infection or the reactivation. Among the induced antibodies, existence of neutralizing antibodies was reported in both young and adult people [35]. Human sera from patients of drug-induced hypersensitivity syndrome (which is highly related with HHV-6 reactivation) and healthy adults were shown to contain antibodies against tetramer (Table 1). This data indicated the antibodies for the tetramer were produced also in the natural infection.

Alum is one of the most commonly used adjuvants for approved vaccines, and it is known to induce humoral immunity via activation in Th2 T cells [31]. Consistently, we observed that the tetramer with Alum induced a significant level of IgG production (Fig 3B). The induced IgG isotypes were dominantly IgG1 (Fig 3C), which indicated Th2 activation. As we used the mouse sera for the neutralizing assay without the heat inactivation of the complements, the complements may have contributed to the enhancement of the neutralization activity. Although the detected amount of IgG2a and IgG3 were relatively low (Fig 3C), they, especially IgG3, possibly contribute to neutralization in a complement-dependent manner more effectively than IgG1. Thus, investigation of the combination of adjuvants to enhance induction of IgG2a and IgG3 isotypes is also important for further developing the tetramer-based vaccine.

D35, an A-class CpG oligodeoxynucleotide adjuvant, has been shown to very effectively induce cellular immunity [28,32]. Here, we also used D35 as an alternative adjuvant, and we expected that it would induce antigen-specific cellular immunity. Moreover, DOTAP and D35 combination has been shown to enhance cellular immunity against the mixed protein antigen [36]. We thus used DOTAP as a delivery tool for the D35 with the tetramer. As expected, the tetramer with D35/DOTAP could greatly induce cellular immunity specific for the tetramer (Fig 4B and 4C). In addition, Alum and D35/DOTAP were observed to induce high humoral immunity for the tetramer, indicating that this combination might be suitable for inducing tetramer-specific immunity, whereas D35/DOTAP could induce high cellular immunity compared to that obtained with Alum (Fig 4B and 4C).

Interestingly, the tetramer itself could also induce the immunity. We therefore examined the innate immunity induced by the tetramer by using human dendritic cells (DCs). Several cytokines/chemokines were induced in the human DCs treated with the tetramer compared to the unstimulated control and treatment with hFc (Fig 7), indicating that the tetramer itself can induce innate immunity.

Since anti-gH and anti-gQ1 mouse antibodies were revealed to have neutralizing potency [20,23,24], antibodies induced by the tetramer could prevent HHV-6B from entering cells by at least two mechanisms. HHV-6B gQ1 is the viral ligand that interacts with the host receptor hCD134 [17]. Therefore, antibodies targeting gQ1 may interrupt the interaction between the viral ligand and the host receptor by masking the receptor binding site on gQ1. An anti-gQ1 monoclonal antibody (KH-1) potently neutralizes *in vitro* HHV-6B infection [24].

On the other hand, anti-gH antibody is unlikely inhibit the receptor recognition. As gH/gL is a regulator of gB, anti-gH antibody may prevent the fusion activity of gH, recognizing the gH fusion domain and/or the communication between gH and gB, but the actual function and the mode of action to regulate gB remain unclear. In addition, antibodies against the tetramer may inhibit its function by interrupting the conformational change in the tetramer, considering the finding that engagement of the viral ligand with the host receptor triggers such a conformational change in many cases [37]. To further investigate the neutralizing mechanism, a structural analysis of the tetramer is necessary.

Herpesviruses have individual surface glycoproteins to recognize and enter their target cells, whereas gH/gL and gB use common machinery. In the development of vaccines against herpesviruses, the viral ligands used for the recognition of the host receptor are attracting attention as candidates for subunit vaccines. gDs of HSV-1 and HSV-2, which function as the viral ligand to recognize the herpesvirus entry mediator (HVEM), and nectin, are major targets of the humoral immunity and the cellular immunity. (reviewed in [38]). HSV-2 gD vaccination has been tried elsewhere [39].

The gH/gL-based glycoprotein complexes of other herpesviruses are also considered prospective candidates for vaccine antigens. For human cytomegalovirus (HCMV), which belongs to the betaherpesvirus subfamily as HHV-6B, the fundamental fusion protein glycoprotein B (gB) has been used as a vaccine antigen with MF59 adjuvant, and the pentametric gH/gL-based complex gH/gL/UL128/UL130/UL131 (pentamer) has also been investigated in response to the demand for more effective vaccines [40]. The pentamer is a determinant of infection to epithelial/endothelial cells by recognizing the receptor Neuropilin 2 [41]. Several studies examined the potential of the pentamer as a vaccine antigen, by mixing with an adjuvant [42] or by expression as a surface antigen of recombinant HCMV [43], alphavirus replication particles [44], or recombinant vaccinia virus ankara [45], and the pentamer demonstrated the strong elicitation of neutralizing antibody against epithelial cell infection, and a less potent elicitation against fibroblast infection.

The Epstein-Barr virus gH/gL/gp42 complex, which recognizes the human leukocyte antigen (HLA) class II on B cells, is recently attracting attention as a candidate EBV vaccine [46], and the development of a vaccine using the glycoprotein 350 (gp350), which recognizes complement receptor 2 (CR2), has been the focus of research so far [47,48]. Because the gH/gL-based complexes have at least two different roles in attachment to specific host receptors and in the subsequent fusion step, the use of the gH/gL-based complex has the advantage of eliciting antibodies to cover the prevention of such broad functions. Although HHV-6B infection depends predominantly on the attachment via the tetramer-hCD134 interaction [17], the tetramer vaccine could prevent the hCD134-independent entry by inducing antibodies against the gH/gL function.

In conclusion, our results revealed that the tetramer, together with the Alum adjuvant, could induce protective humoral immunity for HHV-6B both *in vitro* and *in vivo*. Our results indicate that the tetramer with Alum could be a vaccine candidate for HHV-6B primary infection, whose protection requires humoral immunity. Immunization with D35 and DOTAP could induce cellular immunity for the tetramer, indicating that the combination could be useful for the protection against HHV-6B reactivation, especially in cases of hematopoietic stem cell transplantation. Combinations of distinct adjuvants with the tetramer would be useful as an HHV-6 vaccine strategy for different purposes.

## Methods

### Ethics Statement

All of the animal experimental procedures were approved by the Kobe University Institutional Animal Care and Use Committee (Permission numbers: P141201, P190804, and P150901) and carried out according to the Animal Experimentation Regulations of Kobe University which is based on the Act on Welfare and Management of Animals (Japanese Law No. 105), the Standards Relating to the Care and Management of Laboratory Animals and Relief of Pain (Japanese Ministry of Environment, Notice No. 88, 2006), and the Fundamental Guidelines for Proper Conduct of Animal Experiment and Related Activities in Academic Research Institutions (Japanese Ministry of Education, Culture, Sports, Science and Technology, Notice No. 71, 2006).Concerning the use of CBMCs, this study was approved by the ethical committees of Kobe University Graduate School of Medicine (approval number: No.1026, No.1209 and No.160162). Concerning the use of PBMCs and sera from healthy volunteers, this study was approved by the ethical committees of Kobe University Graduate School of Medicine (approval number: No.1041). Concerning the use of sera from the DIHS patients, this study was approved by the ethical committees of Kobe University Graduate School of Medicine (approval number: No.1358 and No.180070). All the donors were provided informed consent in written form.

### Construction of the mammalian expression plasmids

HHV-6B gH, gL, gQ1, and gQ2 of HST strain were codon-optimized by Invitrogen (https://www.thermofisher.com/jp). The gH fragment for ectodomain expression was amplified and cloned into pFuse-hIgG1-Fc2 plasmid as described previously (InvivoGen, San Diego, CA) [49,50]. The gH and the human IgG1 Fc (hFc) fragments in the resultant plasmid were amplified with the primers containing the sequence for His tag and cloned into pCAGGS-MCS plasmid [51]. We named the resultant plasmid pCAGGS-gHFcHis. The gQ1 full length was amplified and cloned into pCAGGS-MCS plasmid and named as pCAGGS-gQ1.

gQ2 or gL expression cassette was amplified and subcloned into pCAGGS-gQ1 or pCAGGS-gHFcHis plasmid. The resultant plasmids were named pCAGGS-gQ1/gQ2 and pCAGGS-gHFcHis/gL, respectively. Next, puromycin-resistant gene was amplified from pPur plasmid (Clontech, Mountain View, CA) and cloned into pCAGGS-gQ1/gQ2 plasmid, which resulted in the pCAGGS-pur-gQ1/gQ2 plasmid. Neomycin-resistant gene was amplified from pMC1neo-polyA (Agilent Technologies, Waldbronn, Germany) and cloned into pCAGGS-gHFcHis/gL, resulting in the pCAGGS-neo-gHFcHis/gL plasmid. Each ORF has an expression cassette containing the promoter and polyA derived from the pCAGGS plasmid. The ORFs were confirmed by sequence analysis with a 3130 Genetic Analyzer (Applied Biosystems).

### Cells and viruses

HEK293S GnTI^−^ cells were bought from the American Type Culture Collection (ATCC #: CRL-3022). MT4 cells were kindly provided by professor Koyanagi Yoshio from the Kyoto University. Human CD134 (hCD134) expressing JJhan cells were constructed [19] while JJhan cells were obtained from the HHV-6 Foundation Repository (Santa Barbara, CA). MT4 cells and JJhan cells were cultured in RPMI1640 medium (Nissui Pharmaceutical, Tokyo) supplemented with 8% fetal bovine serum (FBS), and HEK293S GnTI^−^ cells were cultured in Dulbecco's Modified Eagle's Medium with 8% FBS (DMEM).

The tetramer-expressing cell line was generated as follows. First, HEK293S GnTI^−^ cells were transfected with the dual-expression plasmids (pCAGGS-pur-gQ1/gQ2 and pCAGGS-neo-gHFcHis/gL) using Lipofectamin2000 (Invitrogen). The transfected cells were cultured in selection medium containing 100 μg/ml of neomycin and 1 μg/ml of puromycin. The viable cells were cloned by endpoint dilution. The single clone of cells expressing four viral glycoproteins were confirmed by an immunofluorescence antibody assay (IFA) using the individual antibodies. The expression was certified by a Western blotting analysis with monoclonal antibodies specific to each component of the tetramer as described previously [24]. The receptor binding activity was evaluated by flow cytometry.

Cord blood mononuclear cells (CBMCs) were stimulated for 72 hr with recombinant human interleukin (IL)-2 (2 ng/ml) and phytohemagglutinin (PHA; 5 μg/ml). HHV-6B strain HST was prepared as described [52]. Concerning the use of CBMCs, this study was approved by the ethical committees of Kobe University Graduate School of Medicine (approval numbers: No.1026, No.1209, and No.160162). Consent was obtained in written form.

### Expression and purification of the tetramer

The tetramer-expressing cell line was cultivated in a chemically defined protein-free medium, CD293 Medium (Thermofisher Scientific, Waltham, MA) supplemented with 1 μg/ml puromycin (InvivoGen) and 20 μg/ml gentamicin at 37°C, 5% CO_2_. The culture supernatant was collected after 2 days, and cell debris was removed by centrifugation (4,000*g*, 4°C, 15 min). The collected medium was supplemented with Na_2_HPO_4_ at a final concentration of 10 mM and then mixed with a 1/100 volume of Ni-NTA agarose (Qiagen) at 4°C for 24 hr. The agarose resin was collected and washed by 20 volumes of a wash buffer (20 mM sodium phosphate buffer pH 7.2, 500 mM NaCl, 20 mM imidazole).

The tetramer was eluted by the elution buffer (20 mM Tris-HCl pH 8.0, 500 mM NaCl, 120 mM imidazole) and concentrated by Amicon Ultra 15 centrifugal filter unit (molecular weight cutoff 50 kDa, Millipore, Bedford, MA). The hFc and His tags were cleaved by adding 10 units of Turbo3c protease (Acceragen, San Diego, CA) per 1 mg protein, and incubated at 4°C for 48 hr. The tetramer and cleaved hFc were further purified by size exclusion column chromatography using a Superdex 200pg column (GE Healthcare, Buckinghamshire, UK) equilibrated with the gel filtration buffer (20 mM Tris-HCl pH 8.0, 100 mM NaCl). The concentration of the tetramer was estimated from the absorbance at 280 nm with the extinction coefficient value 190,555 and the molecular weight 183,545 Da calculated from the amino acid sequence by ProtParam [53].

### Patients and blood sample collection

Serum samples were collected from 14 patients diagnosed as drug-induced hypersensitivity syndrome (DIHS) at Kobe University Hospital between September 2015 and December 2019, along with five healthy volunteers. Presence of HHV-6 DNA in sera from DIHS patients was assessed by nested PCR as previously [54]. Concerning the use of sera from the DIHS patients, this study was approved by the ethical committees of Kobe University Graduate School of Medicine (approval number: No.1358 and No.180070). Concerning the use of sera from healthy volunteers, this study was approved by the ethical committees of Kobe University Graduate School of Medicine (approval number: No.1041). All the donors were provided informed consent in written form.

### Flow cytometry

The hCD134-expressing JJhan cells were first stained with the culture medium of the tetramer expressing HEK293S GnTI^−^ cells or medium as the control at 4°C for 1 hr. After washing, the tetramer was detected by AlexaFluor 488 conjugated goat anti-human IgG at 4°C for 30 min (GE Healthcare). The analysis was performed with a spectral cell analyzer (SA3800, Sony Life Science, San Jose, CA or Tokyo).

### Animal model and vaccination

Three-week-old female BALB/c mice were purchases from CLEA Japan (Tokyo) and the immunodeficient 129S4-*Rag2*^*tm1*.*1Flv*^ *Il2rg*^*tm1*.*1Flv*^ Tg(SIRPA)1Flv/J (hSIRPα-DKO) mice and 129S4-*Rag2*^*tm1*.*1Flv*^ *Il2rg*^*tm1*.*1Flv/J*^ (DKO) mice were obtained from Jackson Laboratory (Bar Harbor, ME, USA). The DKO mice were used for mating to maintain the hSIRPα-DKO strain. All the animals were maintained under specific pathogen-free conditions at the Institute for Experimental Animals at Kobe University Graduate School of Medicine.

For immunization with tetramer vaccine, BALB/c mice (5/group) were immunized by a subcutaneous injection over the shoulders of 200 μl phosphate-buffered saline (PBS) or PBS-containing tetramer mixed with or without the adjuvants including Alum, D35, and DOTAP complexed with D35 on the indicated days. Peripheral blood was taken from the orbital sinus under anesthesia using isoflurane. Euthanasia was performed by the subcutaneous injection with 200 μl pentobarbital.

To generate the hSIRPα-DKO-hCD34 mice, isolated hCD34^+^ hHSPCs were thawed in IMDM containing 10% FBS and 50 μg/mL DNase I (Sigma). Cells were washed twice with PBS. 50,000 to 100,000 hCD34^+^ hHSPCs per mouse were transplanted intravenously into 4–6-week-old hSIRPα-DKO mice after the mice were irradiated twice with total 3.75 Gy [55]. Blood samples were collected at 9–12 weeks post-transplantation, and their peripheral lymphocytes were analyzed by flow cytometry.

### Measurements of antibody titers for HHV-6 tetramer

Mouse serum samples were collected after 30 min incubation at 37°C and collected by centrifugation. The presence of antibodies against the HHV-6 tetramer was tested by an enzyme-linked immunosorbent assay (ELISA). For the ELISA, plates were coated overnight with 0.5 μg/ml purified HHV-6B tetramer in 0.1 M carbonate-bicarbonate buffer at 4°C, washed with 0.05% Tween-20 in PBS (PBST), and blocked with 0.25% bovine serum albumin (BSA) in PBS. Serial dilutions of sera in 0.05% BSA and 0.05% Tween-20 in PBS were incubated at 37°C for 1 hr on the plates. The plates were washed with PBST and added with horseradish-peroxidase (HRP)-conjugated anti-mouse IgG (GE Healthcare). After incubation, proteins were detected by ABTS solution (Roche) and the absorbance was measured at 405 nm with a Multimode reader TriStar LB941 (Berthold). For the measurement of IgG1, IgG2a and IgG3, the first antibodies were diluted to 1:2560 and the secondary antibodies were added to HRP-conjugated anti-mouse IgG1, IgG2a or IgG3 (Abcam, Cambridge, UK). Isotype control antibodies of mouse IgG1, IgG2a, and IgG3 (MBL, Japan) were coated on the plate and used to make standard curves to translate from the OD405 values to the antibody amount. The anti-tetramer antibody titers of the sera from DIHS patients and healthy volunteers were measured by the same procedures as mouse serum while the HRP-conjugated anti-human IgG antibody (GE Healthcare) were used instead of anti-mouse IgG antibody.

### Measurements of antibody titers for HHV-6B infected cells

To detect the antibody titers against HHV-6B infected cells, MT4 cells were infected with the HHV-6B virus (HST strain) and prepared for the indirect immunofluorescence assay (IFA) 3 days post-infection as described previously [24]. In brief, serial two-fold dilutions of the sera from DIHS patients and healthy volunteers were used as the first antibody followed by the staining of AlexaFluor 488 conjugated goat anti-human IgG. The reciprocal of the dilution at which no fluorescent signal appeared was regarded as the antibody titer. Uninfected MT4 cells were used as a negative control.

### Virus neutralization assay

Neutralization assays were performed as described [24]. Sera used for virus neutralization assay were not heat inactivated. Serial twofold dilutions of mouse sera were incubated with HHV-6B (HST strain) virus solution at 37°C for 30 min. The virus-serum mixed solutions were then added to MT4 cell pellets and incubated at 37°C for 1 hr. The remaining virus was removed by washing with fresh medium, and the cells were cultured for 4 days. IE1 expression was detected by an indirect immunofluorescence assay (IFA) as described [10,24].

### Tetramer stimulation and the cytokine ELISA assay

Single cell suspensions were prepared from the mouse spleens by dissociation and passing through a cell strainer. Red blood cells were removed by treatment with hemolysis buffer (150 mM NH_4_Cl, 10 mM KHCO_3_, 0.1 mM EDTA) for 5 min, and the remaining splenocytes from the same group were mixed together and washed for the depletion experiment. CD4^+^ or CD8^+^ T cells were removed using a CD4 or CD8 micro-beads set with MACS manual separators and columns (Miltenyi Biotec, Bergisch-Gladbach, Germany) following the standard protocols.

Depleted cells, along with the non-depleted controls, were prepared as 2 × 10^6^ cells per well cultured in RPMI supplemented with 10% FBS in a 96-well plate. Next, 0.1 μg of purified tetramer or the same amount of hFc was added to one well. We also used 5 μg of the peptide of gQ1, p43 [27] for the stimulation. Supernatants of the cultured cells were collected 24 hr later. Mouse IFN-γ and IL-13 were measured using IFN-γ and IL-13 Mouse ELISA kits (Thermo Fisher Scientific) following the standard protocols.

### Infection in humanized mice

To prepare the infection, numbers of human T cells were expanded by intraperitoneal injection of the prepared CBMCs into hSIRPα-DKO-hCD34 mice as mentioned previously [33]. The portion of human CD3-positive cells was monitored by flow cytometry every week after the transfer of CBMCs as described previously [33]. The time point for infection was determined as the percentage of hCD3^+^ cells among human CD45^+^ cells was >10% and the ratio of CD3^+^ T lymphocytes in hCD45^+^ cells was >90% [33]. Before infection, 200 μl of the sera from the tetramer-immunized mice with determined neutralizing titers or only adjuvants immunized mice were intravenously injected into humanized mice. The next day, all the mice were intravenously injected with 200 μl of the cell-free HHV-6B virus (9.6×10^4^ TCID 50/ml titered in MT4 cells). Organs were collected from the anesthetized mice 7 days post-infection.

### Viral detection in humanized mice

Genomic DNAs were extracted from the tissues using a QIAGEN DNeasy Blood & Tissue kit following the manufacturer's protocol (Qiagen). To detect the viral genome DNAs, a quantitative real-time PCR was performed as described [56]. Formalin-fixed sections for the detection of viral proteins were prepared and stained as described previously [33]. Briefly, formalin-fixed and paraffin-embedded (FFPE) tissue blocks were sectioned at 4 μm and stained with either of mouse monoclonal antibody against human CD3ε (clone F7.2.38) purchased from Dako (Glostrup, Denmark) at 1:50 and the rabbit antiserum against HHV-6B IE1 (1:2,000). To prevent the non-specific binding, Mouse-to-Mouse Blocking Reagent (ScyTek Laboratories, Inc., UT, USA) was used before the staining of secondary antibody. The immunostaining process was performed using a Bond Max autostainer (Leica Microsystems, Wetzlar, Germany).

### Generation of monocyte-derived dendritic cells

Monocytes were isolated from the PBMCs of healthy volunteers by Ficoll-Conray density gradient centrifugation. The monocytes were cultured in RPMI 1640 medium supplemented with 2% fetal bovine serum (FBS) at 37°C. One hour later, the non-adherent cells and adherent cells were isolated, and each type of cell was separately cultured in fresh RPMI 1640 medium supplemented with 10% FBS, 800 U/ml human recombinant granulocyte-macrophage colony-stimulating factor (GM-CSF), and 400 U/ml IL-4 (R&D Systems). At 4 days after the initiation of the culture, the medium was completely replaced with fresh medium. Seven days after the initiation of the culture, the adherent cells were collected for stimulation. Concerning the use of PBMCs from healthy volunteers, this study was approved by the ethical committees of Kobe University Graduate School of Medicine (approval number: No.1041). All the donors were provided informed consent in written form.

### Human monocyte-derived DCs stimulated with tetramer

Human monocyte-derived DCs were seeded at 1×10^6^ cells per well and stimulated with 50 μg of HHV-6B tetramer. The same amount of purified hFc was used as the control along with the unstimulated group, adding the same amount of medium. After being mixed, the cells were incubated at 4°C for 30 min and then at 37°C for 40 min. The medium was then washed three times, and the cells were cultured for 24 hr before the supernatant was collected. Samples were stored at −80°C for the subsequent analysis.

### The measurement of chemokines by the Bio-Plex system

To analyze the production of chemokines, we subjected the culture supernatants of human monocyte-derived DCs stimulated by culture medium, hFc, or tetramer to a Bio-Plex Pro Human Chemokine Assays 40-plex kit (Bio-Rad) that contains 6Ckine/CCL21, BCA-1/CXCL13, CTACK/CCL27, ENA-78/CXCL5, Eotaxin/CCL11, Eotaxin-2/CCL24, Eotaxin-3/CCL26, Fractalkine/CX3CL1, GCP-2/CXCL6, GM-CSF, Gro-α/CXCL1, Gro-β/CXCL2, I-309/CCL1, IFN-γ, IL-1β, IL-2, IL-4, IL-6, IL-8/CXCL8, IL-10, IL-16, IP-10/CXCL10, I-TAC/CXCL11, MCP-1/CCL2, MCP-2/CCL8, MCP-3/CCL7, MCP-4/CCL13, MDC/CCL22, MIF, MIG/CXCL9, MIP-1α/CCL3, MIP-1δ/CCL15, MIP-3α/CCL20, MIP-3β/CCL19, MIPF-1/CCL23, SCYB16/CXCL16, SDF-1α+β/CXCL12, TRAC/CCL17, TECK/CCL25, and TNF-α as magnetic beads with antibody (Bio-Rad). The experiment was performed according to the kit's protocol.

### Statistical analyses

Statistical differences were determined by Student's t test and p<0.05 was considered statistically significant.

## Supporting information

S1 Fig Plot for the human sera binding to the tetramer in the ELISA. The OD405 values per fold dilution of each sera from (A) DIHS patients and (B) healthy adults were plotted. The cutoff value 0.1 (red line) was determined considering the signal of the No 4 at the 10- fold dilution in the (A). The determined titer of each plot was indicated by a red arrow.
(TIF)
