# Supplementary figures and images for: Tetrameric glycoprotein complex gH/gL/gQ1/gQ2 is a promising vaccine candidate for human herpesvirus 6B
Source: PLoS Pathog. 2020 Jul 23;16(7):e1008609. doi: 10.1371/journal.ppat.1008609 (PMC7377363; doi:10.1371/journal.ppat.1008609)

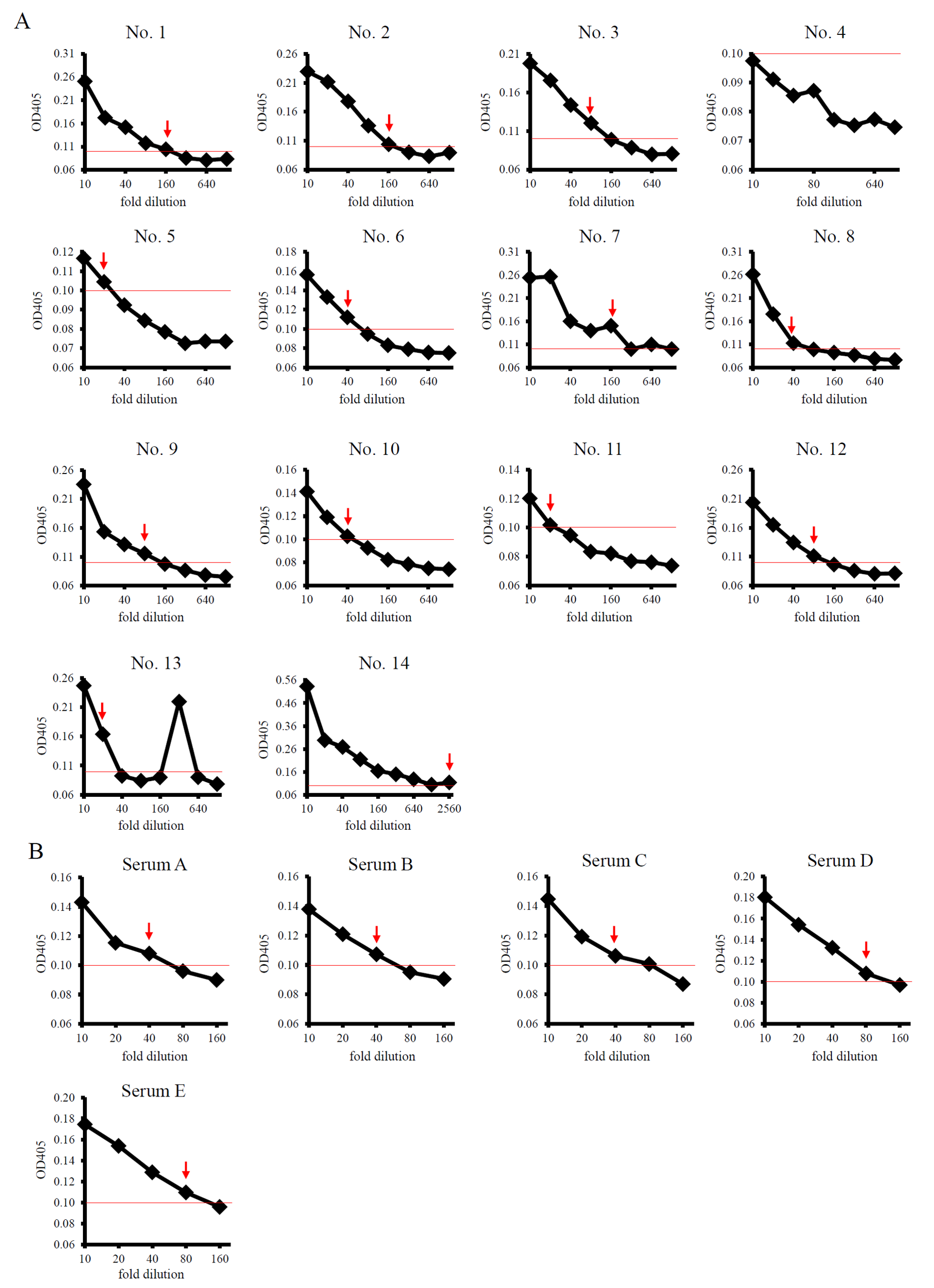

Supplement: S1 Fig — The OD405 values per fold dilution of each sera from (A) DIHS patients and (B) healthy adults were plotted. The cutoff value 0.1 (red line) was determined considering the signal of the No 4 at the 10- fold dilution in the (A). The determined titer of each plot was indicated by a red arrow. (TIF) [file ppat.1008609.s001.tif]
